# Supplementary material for: Promoting Drp1-mediated mitochondrial fission in midlife prolongs healthy lifespan of Drosophila melanogaster
Source: Nat Commun. 2017 Sep 6;8:448. doi: 10.1038/s41467-017-00525-4 (PMC5587646; doi:10.1038/s41467-017-00525-4)
Supplement: Supplementary file 1 — Supplementary Information [file 41467_2017_525_MOESM1_ESM.pdf]

Description of Supplementary Files

File Name: Peer Review File

File Name: Supplementary Information
Description: Supplementary Figures, Supplementary Table.

Supplementary Figure 1. Midlife Drp1 induction extends lifespan

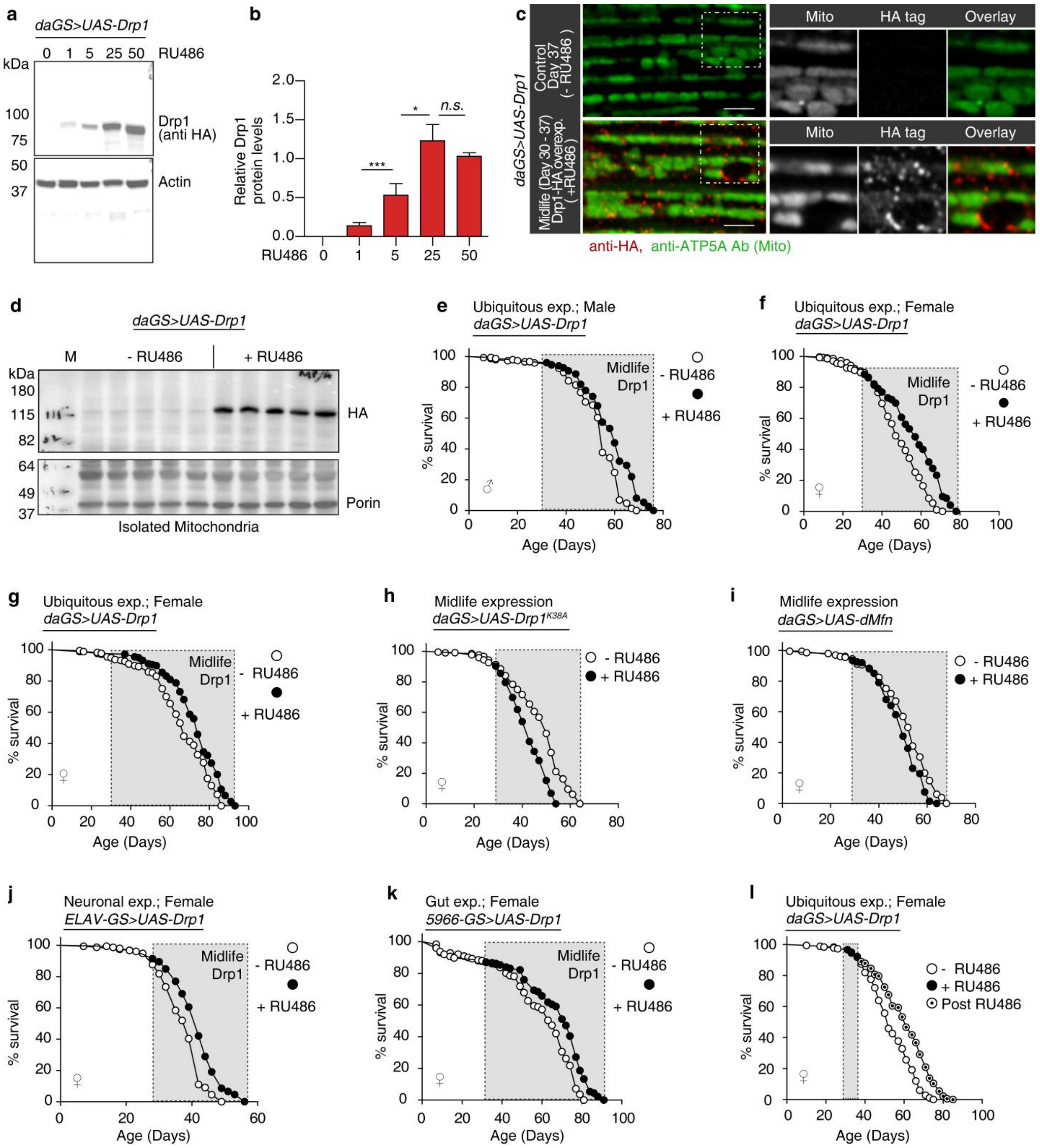

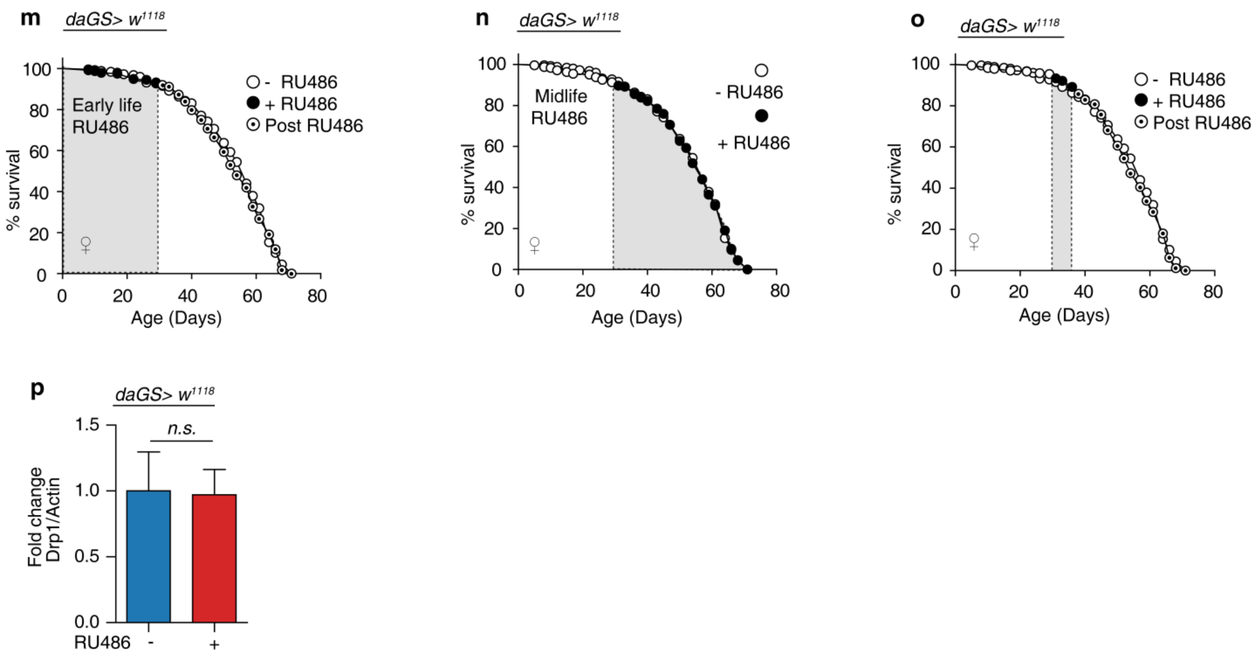

(a-b) Western blot (a) detection of Drp1 from day 37 *daGS>UAS-Drp1* females with or without RU486-mediated transgene induction from day 30 onwards. RU486 was provided in the media at a concentration of 1, 5, 25 and 50µg/ml. Densitometry of blots (b). n = 4 biological replicates with 8 individual flies per replicate; ***p < 0.001 and *p < 0.05; one-way ANOVA/Bonferroni's multiple comparisons test.

(c) Immunostaining of indirect flight muscles from 37 day old *daGS>UAS-Drp1-HA* females with or without RU486-mediated transgene induction for 7 days from day 30 to day 37, showing mitochondria (green channel, anti ATP5a) and Drp1 (red channel, anti HA). Scale bar is 5µm.

(d) Western blot detection of HA levels in the mitochondrial fraction isolated from day 37 *daGS>UAS-Drp1* females with or without RU486-mediated transgene induction from day 30 onwards.

(e) Survival curves of *daGS>UAS-Drp1* males with or without RU486-mediated transgene induction from day 30 onwards. The shaded area indicates the duration of Drp1 induction. $p<0.0001$, log rank test; $n > 146$ flies.

(f) Survival curves of *daGS>UAS-Drp1* females with or without RU486-mediated transgene induction from day 30 onwards. The shaded area indicates the duration of Drp1 induction. $p<0.0001$, log rank test; $n > 270$ flies.

(g) Survival curves of *daGS>UAS-Drp1* (another independently generated transgene) females with or without RU486-mediated transgene induction from day 30 onwards. The shaded area indicates the duration of Drp1 induction. $p<0.0001$, log rank test; $n > 177$ flies.

(h) Survival curves of *daGS >UAS-Drp1*^{K38A} females with or without RU486-mediated transgene induction from day 30 onwards. The shaded area indicates the duration of Drp1 induction. $p<0.0001$, log rank test; $n > 208$ flies.

(i) Survival curves of *daGS >UAS-dMfn* females with or without RU486-mediated transgene induction from day 30 onwards. The shaded area indicates the duration of Drp1 induction. $p<0.0001$, log rank test; $n > 181$ flies.

(j) Survival curves of *ELAV-GS >UAS-Drp1* females with or without RU486-mediated transgene induction from day 30 onwards. The shaded area indicates the duration of Drp1 induction. $p<0.0001$, log rank test; $n > 152$ flies.

(k) Survival curves of *5966GS>UAS-Drp1* females with or without RU486-mediated transgene induction from day 30 onwards. The shaded area indicates the duration of Drp1 induction. $p<0.0001$, log rank test; $n > 225$ flies.

(l) Survival curves of *daGS>UAS-Drp1* females with or without RU486 feeding from day 30 to day 37. The shaded area indicates the duration of RU486 feeding. $p<0.0001$, log rank test; $n > 182$ flies.

(m) Survival curves of *daGS>*w^{1118} females with or without RU486 feeding from day 1 to day 30. The shaded area indicates the duration of RU486 feeding. $P>0.05$, log rank test; $n > 236$ flies.

(n) Survival curves of *daGS>*w^{1118} females with or without RU486 feeding from day 30 onwards. The shaded area indicates the duration of RU486 feeding. $P>0.05$, log rank test; $n > 241$ flies.

(o) Survival curves of *daGS>*w^{1118} females with or without RU486 feeding from day 30 to day 37. The shaded area indicates the duration of RU486 feeding. $P>0.05$, log rank test; $n > 239$ flies.

(p) QPCR analyses of *Drp1* mRNA levels on day 37 in *daGS>*w^{1118} females with or without RU486 feeding from day 30 to day 37. $n = 5$ replicates with 3 flies per replicate; $p > 0.05$ and is non-significant (*n.s.*); two-tailed unpaired t-test.

Bars (b and p) depict mean ± S.D.

Supplementary Figure 2. RU486 does not impact healthspan in control flies and inhibiting mitochondrial fission/promoting mitochondrial fusion confers early-onset intestinal barrier dysfunction

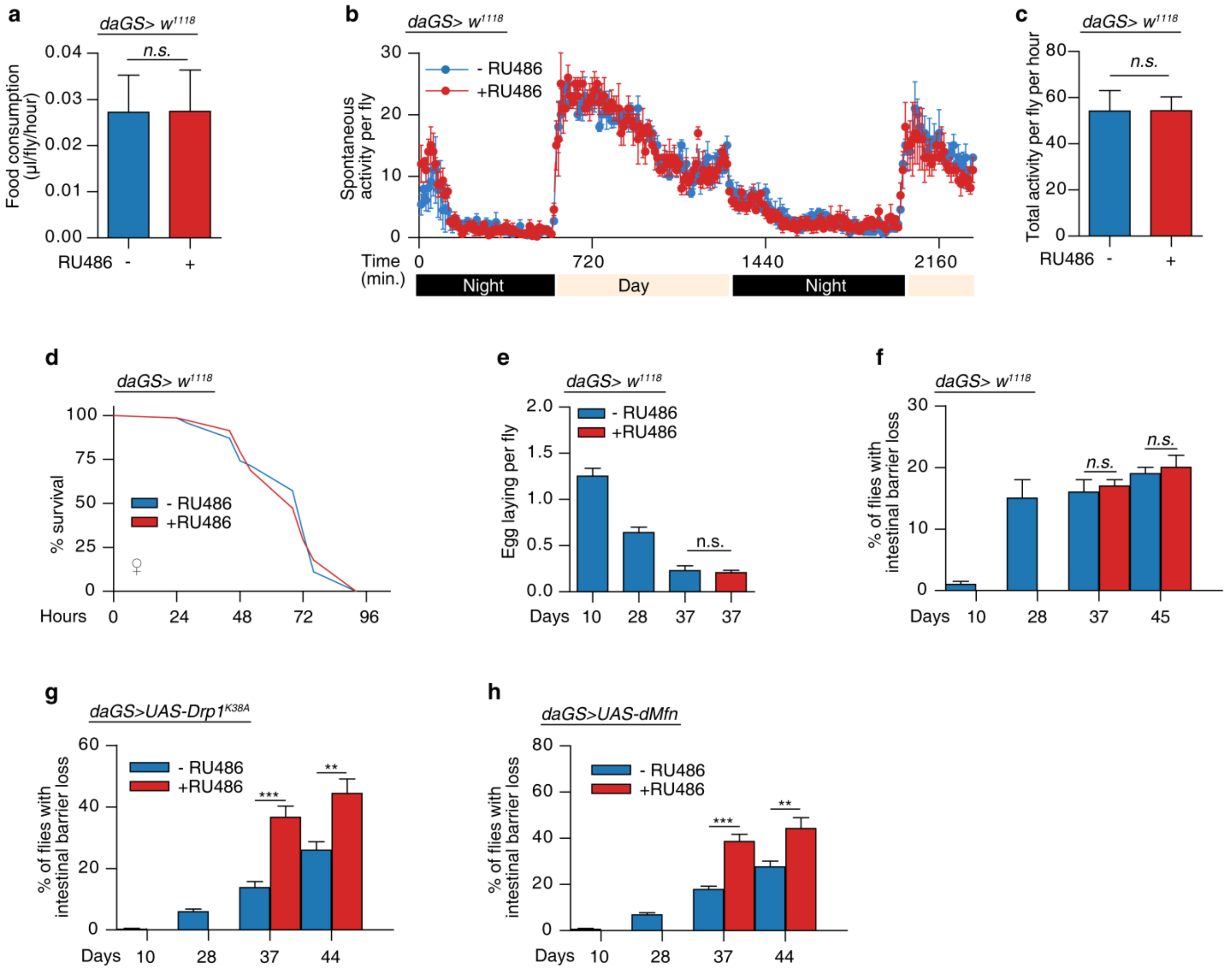

(a) Capillary feeding assay (CAFE) of 37 day old *daGS>w*^{1118} females with or without RU486 feeding. n = 8 vials of 10 flies per condition; p > 0.05 and is non-significant (*n.s.*); two-tailed unpaired t-test.

(b and c) Spontaneous activity graph (b) of 37 day old *daGS>w*^{1118} females with or without RU486 feeding as measured by DAM system beam breaks. Quantification of total activity per

fly per hour (c) from spontaneous activity graphs. n = 3 vials of 10 flies per condition; $p > 0.05$ and is non-significant (*n.s.*); two-tailed Mann Whitney U test.

(d) Starvation survival curves of *daGS>w*^{1118} females with or without RU486 feeding from day 30 onwards. $p > 0.05$; log rank test; n = 100 flies.

(e) Fecundity time course of *daGS>w*^{1118} females with or without RU486 feeding since day 30 onwards. n = 360 flies on day 10; $p > 0.05$ and is non-significant (*n.s.*); one-way ANOVA/Bonferroni's multiple comparisons test.

(f) Intestinal integrity (Smurf) assay during aging of *daGS>w*^{1118} females with or without RU486 feeding since day 30 onwards. n = 292 flies on day 10; $p > 0.05$ and is non-significant (*n.s.*); one-way ANOVA/Bonferroni's multiple comparisons test.

(g) Intestinal integrity (Smurf) assay during aging of *daGS >UAS-Drp1*^{K38A} females with or without RU486 feeding since day 30 onwards. n = 360 flies on day 10; ***$p < 0.001$ and **$p < 0.01$; one-way ANOVA/Bonferroni's multiple comparisons test.

(h) Intestinal integrity (Smurf) assay during aging of *daGS >UAS-dMfn* females with or without RU486 feeding since day 30 onwards. n = 360 flies on day 10; ***$p < 0.001$ and **$p < 0.01$; one-way ANOVA/Bonferroni's multiple comparisons test.

Bars (a and c) depict mean ± S.D and bars (e, f, g and h) depict mean ± S.E.M.

Supplementary Figure 3. RU486 does not impact mitochondrial morphology or TMRE fluorescence in aged control flies

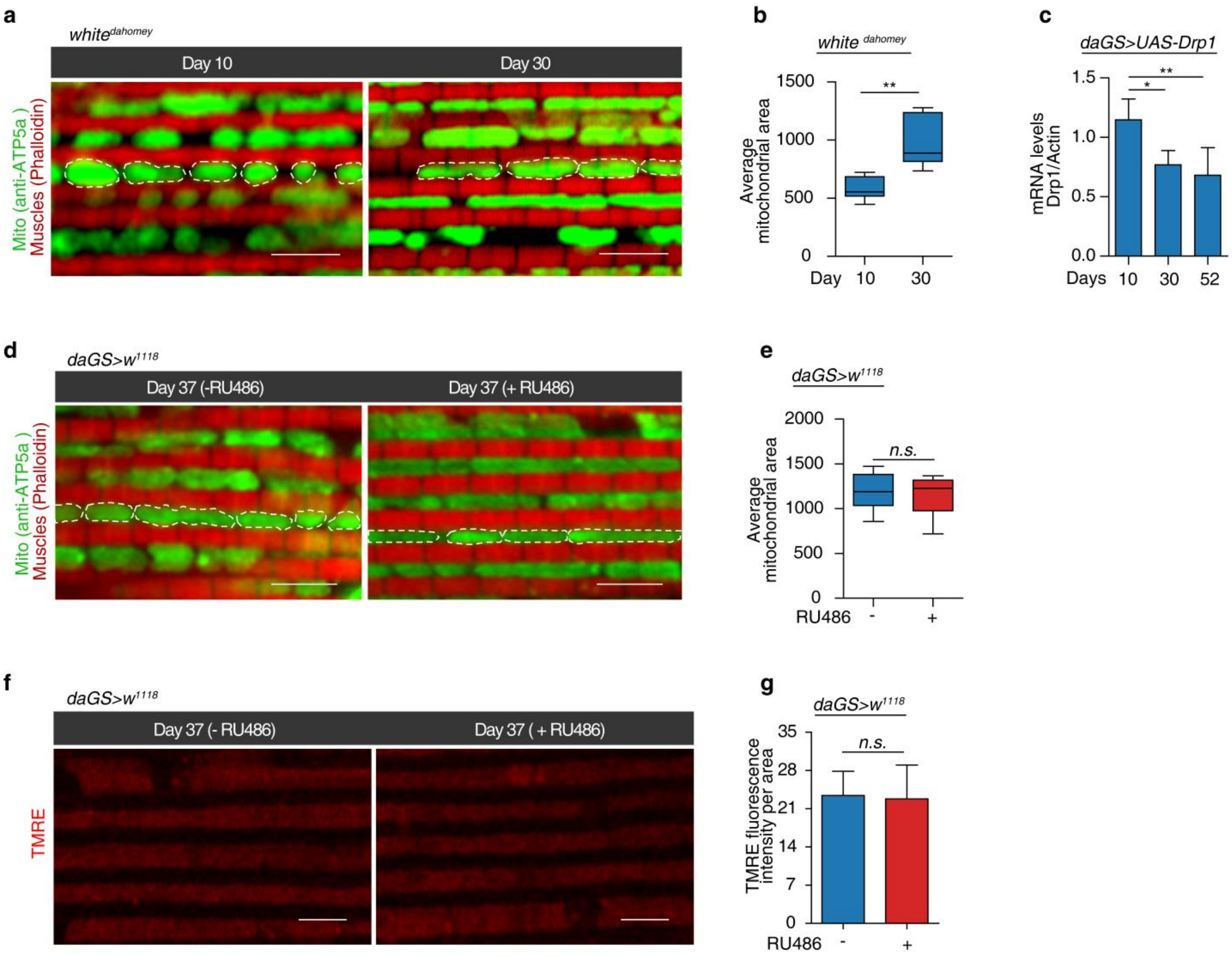

(a and b) Immunostaining of indirect flight muscles (a) from 10 and 30 day old *white*^{dahomey} females showing mitochondria (green channel, anti ATP5a) and muscles (red channel, stained with phalloidin/F-actin). Scale bar is 5μm. Quantification of mitochondrial size (b); n = 7; **$p < 0.01$; two-tailed unpaired t-test.

(c) qPCR analyses of *Drp1* mRNA levels on day 10, 30 and 52 in *daGS>UAS-Drp1* females; n = 5 replicates with 3 flies per replicate; $**p < 0.01$ and $*p < 0.05$; one-way ANOVA/Bonferroni's multiple comparisons test.

(d and e) Immunostaining of indirect flight muscles (d) from day 37 *daGS>*w^{1118} females with or without RU486 feeding for 7 days from day 30 to day 37 showing mitochondria (green channel, anti ATP5a) and muscles (red channel, rhodamine staining for F-actin). Scale bar is 5µm. Quantification of mitochondrial size (e); n = 8; $p > 0.05$ is non-significant (*n.s.*); two-tailed unpaired t-test.

(f and g) Indirect flight muscles (f) from day 37 *daGS>*w^{1118} females with or without feeding from day 30 onwards showing TMRE staining as a marker for mitochondrial membrane potential. Quantification of mitochondrial membrane potential (g) measured by TMRE staining as shown in (f). n = 8-12 flies; $p > 0.05$ is non-significant (*n.s.*) ; two-tailed unpaired t-test.

Boxplots (b and e) display the first and third quartile, with the horizontal bar at the median and whiskers showing the most extreme data point, which is no more than 1.5 times the interquartile range from the box. Bars (c and g) depict mean ± S.D.

Supplementary Figure 4. RU486 does not impact mitochondrial complex I activity or ROS levels in control flies

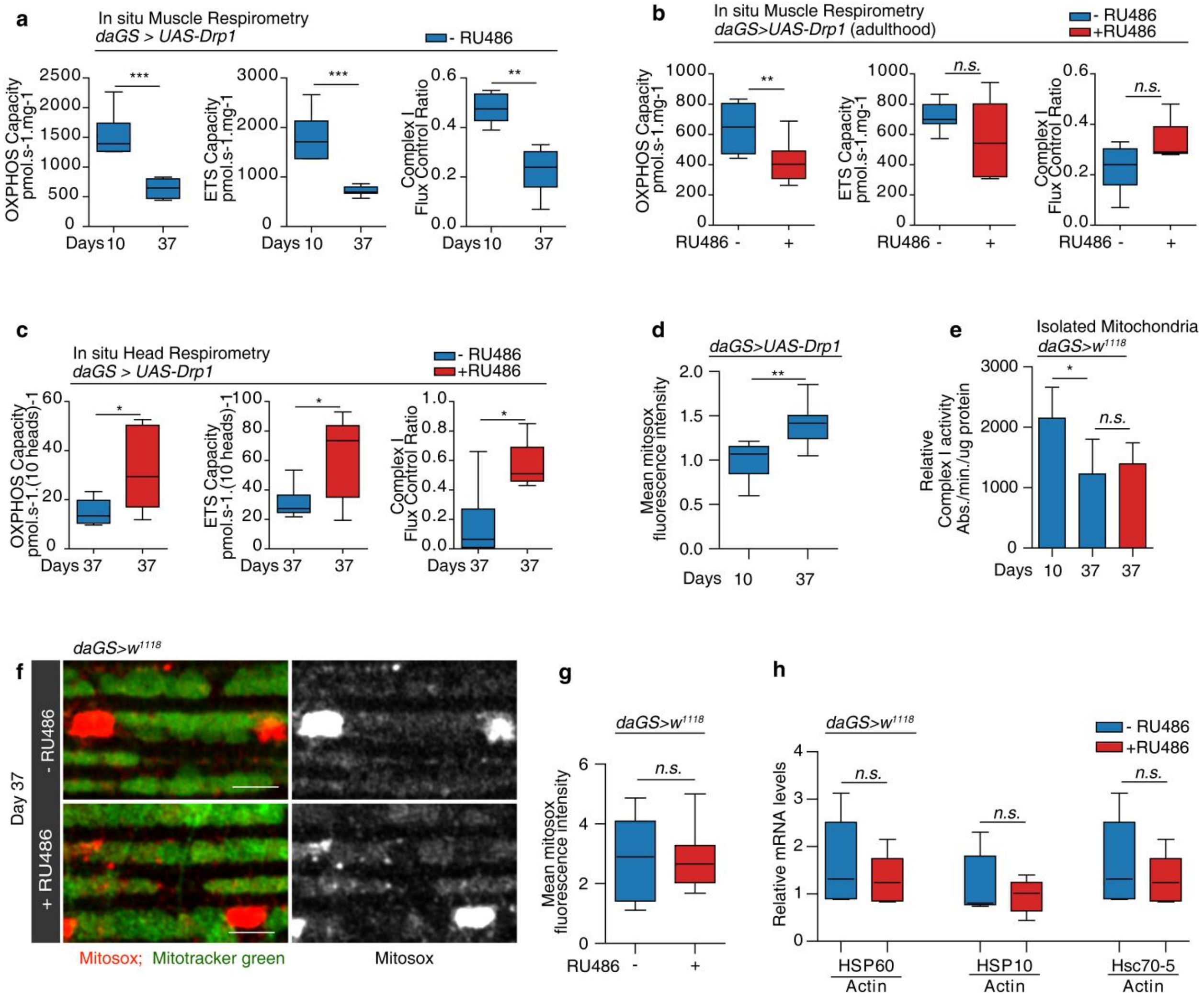

(a) *In situ* respirometry of permeabilized muscle bundles from 10 and 37 day old *daGS>UAS-Drp1* females to assess the capacity for oxidative phosphorylation (OXPHOS) and Electron Transport System (ETS) flux, and the flux control ratio of Complex I by rotenone inhibition. n = 6-8 replicates with 2 thoraces per replicate; ***p < 0.001 and **p < 0.01; two-tailed unpaired t-test.

(b) *In situ* respirometry of permeabilized muscle bundles from 37 day old *daGS>UAS-Drp1* females with or without RU486 feeding from day 3 onwards to assess the capacity for oxidative phosphorylation (OXPHOS) and Electron Transport System (ETS) flux, and the flux control ratio of Complex I by rotenone inhibition. n = 6-8 replicates with 2 thoraces per replicate; $p > 0.05$ is non-significant (*n.s.)*; two-tailed unpaired t-test.

(c) *In situ* respirometry of permeabilized heads from 37 day old *daGS>UAS-Drp1* females to assess the capacity for oxidative phosphorylation (OXPHOS) and Electron Transport System (ETS) flux, and the flux control ratio of Complex I by rotenone inhibition. n = 5-6 replicates with 10 heads per replicate; $^*p < 0.05$; two-tailed unpaired t-test.

(d) Quantification of free superoxide radicals from staining of indirect flight muscles from 10 and 37 day old *daGS>UAS-Drp1* females. Staining was done for mitochondria (Mitotracker green staining) and levels of superoxide radicals (staining with MitoSOX™ reagent oxidation of which produce red fluorescence when it interacts with superoxide radicals). n = 9 replicates; $p > 0.05$ is non-significant (*n.s.*); two-tailed unpaired t-test.

(e) Quantification of marker of mitochondrial activity in 10 and 37 day old *daGS>*w^{1118} females with or without RU486 feeding for 7 days from day 30 to day 37. Complex I activity measurement in isolated mitochondrial pellet from 10 and 37 day old adult females. n = 5 replicates with 8 flies per replicate; $^*p < 0.05$ and $p > 0.05$ is non-significant (*n.s.)*; one-way ANOVA/Bonferroni's multiple comparisons test.

(f and g) Staining of indirect flight muscles (f) from 37 day old *daGS>w^{1118}* females with or without RU486 feeding for 7 days from day 30 to day 37, showing mitochondria (green channel, Mitotracker green staining) and levels of superoxide radicals (red channel, staining with MitoSOX™ reagent oxidation of which produce red fluorescence when it interacts with superoxide radicals). Scale bar is 5µm. Quantification of free superoxide radicals (g); n = 8-9 flies; $p > 0.05$ is non-significant (*n.s.*); two-tailed unpaired t-test.

(h) qPCR analyses of *Hsp60*, *Hsp10* and *mtHsp70* (*Hsc70-5*) on day 37 in *daGS*> w^{1118} females with or without RU486 feeding midlife (day 30) onwards. n = 5 replicates with 3 flies per replicate; $p > 0.05$ and is non-significant (*n.s.*); two-tailed unpaired t-test.

Boxplots (a-d, g and h) display the first and third quartile, with the horizontal bar at the median and whiskers showing the most extreme data point, which is no more than 1.5 times the interquartile range from the box. Bars (e) depict mean ± S.D.

Supplementary Figure 5. RU486 does not impact proteostasis in aged control flies

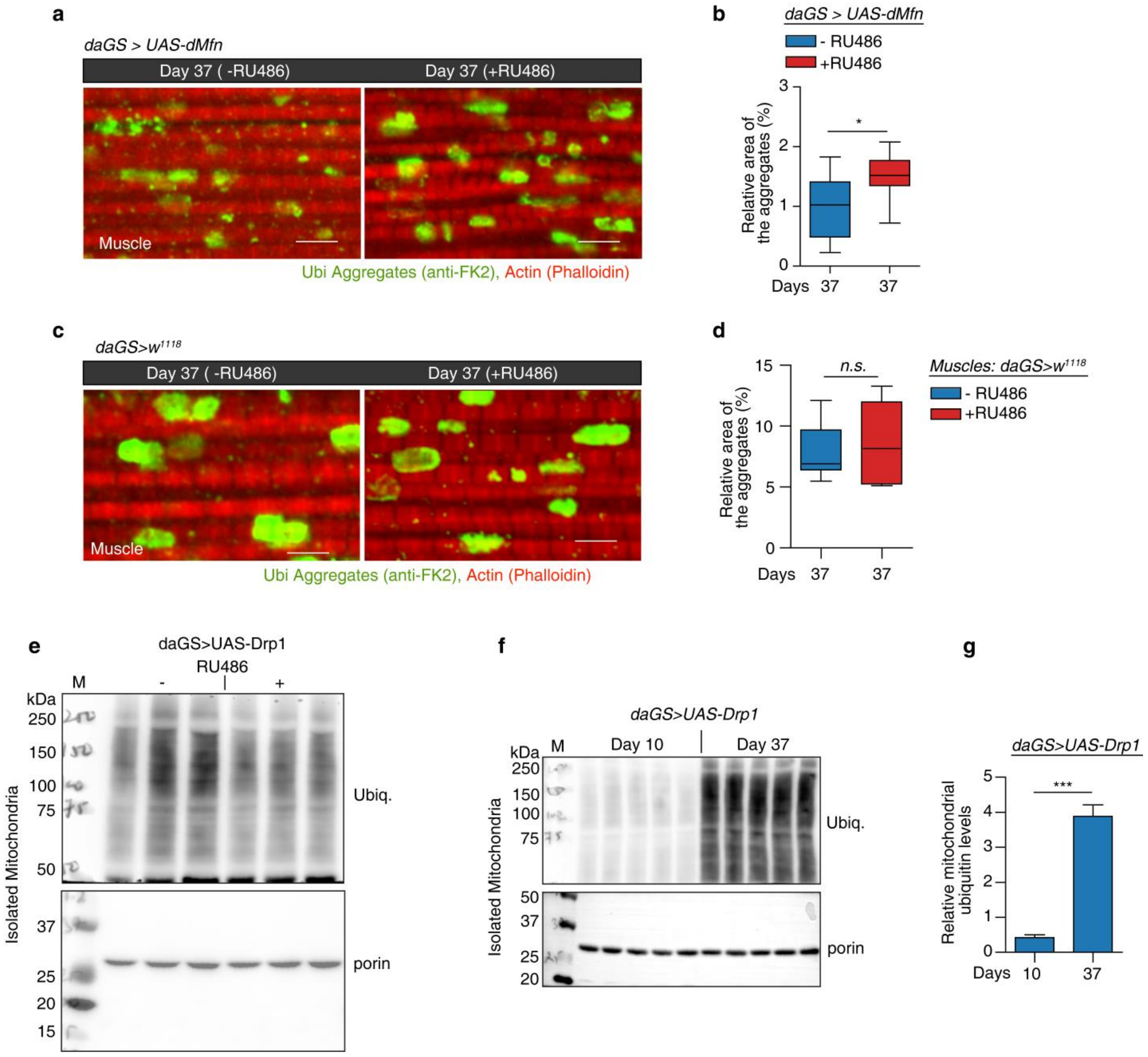

(a) Immunostaining of indirect flight muscles from day 37 *daGS>dMfn* females with or without RU486 feeding from day 30 onwards showing protein polyubiquitinated aggregates (red channel, muscles stained with phalloidin/F-actin and green channel, antipolyubiquitin). Scale bar is 5µm.

(b) Quantification of polyubiquitin aggregates in muscle (as shown in a); n = 9-14 flies; *$p < 0.05$; two-tailed unpaired t-test.

(c) Immunostaining of indirect flight muscles from day 37 *daGS*> w^{1118} females with or without RU486 feeding from day 30 onwards showing protein polyubiquitinated aggregates (red channel, muscles stained with phalloidin/F-actin and green channel, antipolyubiquitin). Scale bar is 5µm.

(d) Quantification of polyubiquitin aggregates in muscle (as shown in c); n = 7-9 flies; $p > 0.05$ and is non-significant (*n.s.*); two-tailed Mann Whitney U test.

(e) Western blot detection of total ubiquitin-conjugated proteins in isolated mitochondria from day 37 *daGS>UAS-Drp1* females with or without RU486-mediated transgene induction from day 30 to day 37.

(f-g) Western blot (f) detection of total ubiquitin-conjugated proteins in isolated mitochondria from day 10 and 37 *daGS>UAS-Drp1* females. Densitometry of ubiquitin blots (g) from mitochondrial pellet; n = 6 replicates, 25 flies per replicate; ***$p < 0.001$; two-tailed unpaired t-test.

Boxplots (b and d) display the first and third quartile, with the horizontal bar at the median and whiskers showing the most extreme data point, which is no more than 1.5 times the interquartile range from the box. Bars (g) depict mean ± S.D.

Supplementary Figure 6. Midlife Drp1 induction facilitates mitophagy in aged flies

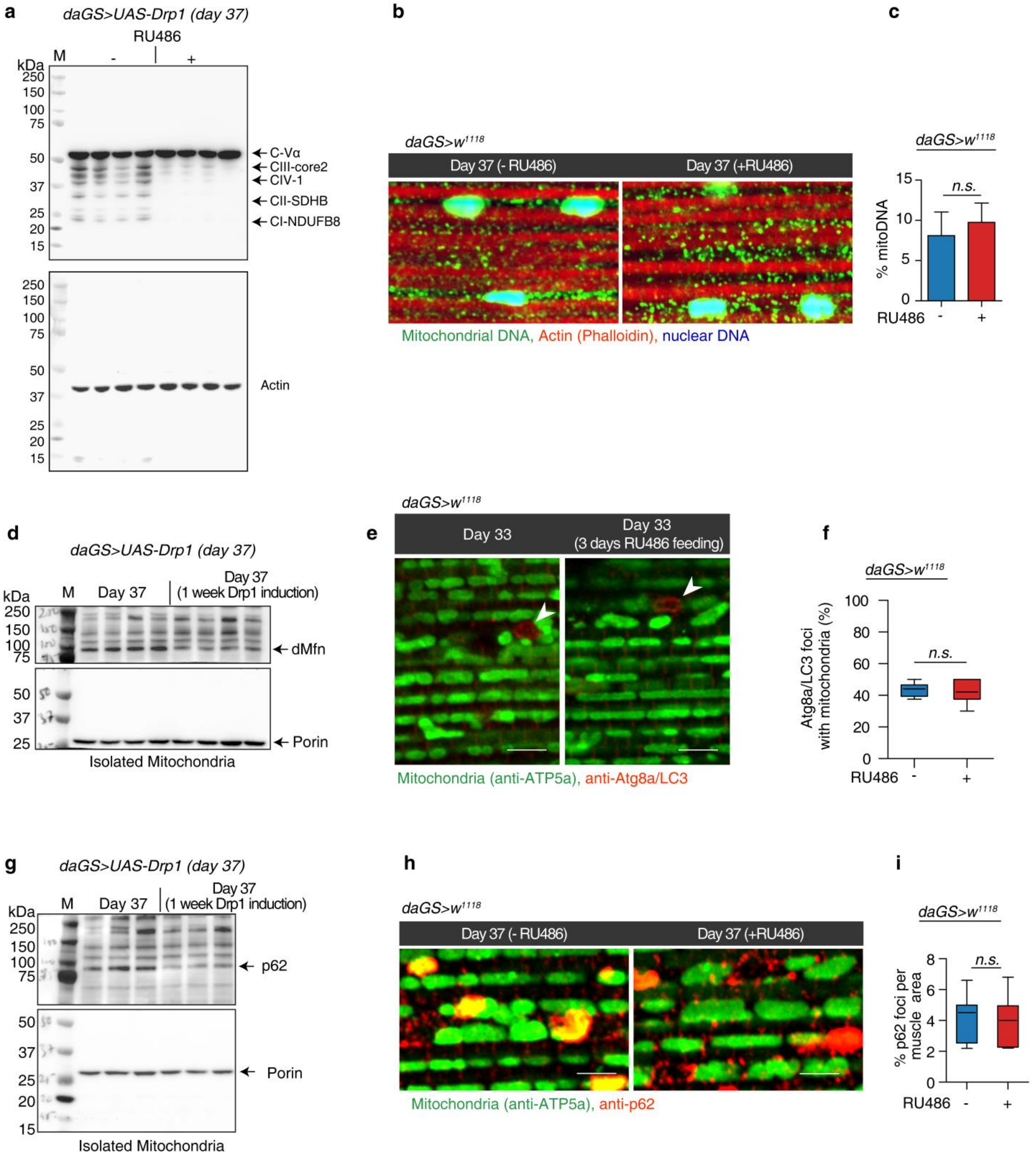

(a) Western blot detection of mitochondrial respiratory complex subunits in thoraces isolated from day 37 *daGS>UAS-Drp1* females with or without RU486-mediated transgene induction

from day 30 to day 37. Quantification for each specific protein is indicated in ratios between with or without RU486-mediated transgene induction from day 30 to day 37. For C-Vα, CIII-core2, CIV-1, CII-SDHB, and CI-NDUFB8 the ratios are 1:0.96, 1:0.21, 1:0.12, 1:0.06, and 1:0.06 respectively.

(b and c) Immunostaining of indirect flight muscles (b) from day 37 *daGS*> w^{1118} females with or without RU486 feeding from day 30 to day 37, showing mitochondrial DNA (green channel, anti-ds DNA antibody), nuclear DNA (blue channel, stained with TO-PRO-3) and muscles (red channel, stained with phalloidin/F-actin). Scale bar is 5µm. Quantification of mitochondrial ds-DNA (c) in muscles (as shown in b); n = 6 flies; p > 0.05 and is non-significant (*n.s.*); two-tailed unpaired t-test.

(d) Western blot detection of mitochondrial fusion-promoting factor Mitofusin in isolated mitochondria from day 37 *daGS>UAS-Drp1* females with or without RU486 feeding from day 30 to day 37.

(e-f) Immunostaining of indirect flight muscles (e) from day 37 *daGS*> w^{1118} females with or without RU486 feeding from day 30 to day 37 showing mitochondria (green channel, anti ATP5a) and an autophagic marker (red channel, anti-ATG8a). Scale bar is 5µm. Quantification (f) of ATG8a foci co-localizing with mitochondria (as shown in e); n = 6 flies; p > 0.05 and is non-significant (*n.s.*); two-tailed unpaired t-test.

(g) Western blot detection of P62 levels in isolated mitochondria from day 37 *daGS>UAS-Drp1* females with or without RU486-mediated transgene induction from day 30 to day 37.

(h-i) Immunostaining of indirect flight muscles (h) from day 37 *daGS*> w^{1118} females with or without RU486 feeding from day 30 to day 37, showing mitochondria (green channel, anti ATP5a) and p62 (red channel, anti-p62). Scale bar is 5µm. Quantification (i) of P62 foci per muscle area (as shown in h); n = 7-8 flies; $p > 0.05$ and is non-significant (*n.s.*); two-tailed unpaired t-test.

Bars (c) depict mean ± S.D. Boxplots (f and i) display the first and third quartile, with the horizontal bar at the median and whiskers showing the most extreme data point, which is no more than 1.5 times the interquartile range from the box.

Supplementary Figure 7. Midlife RNAi of Atg1 is required for *dMfn*-mediated longevity but does not impact lifespan in control flies

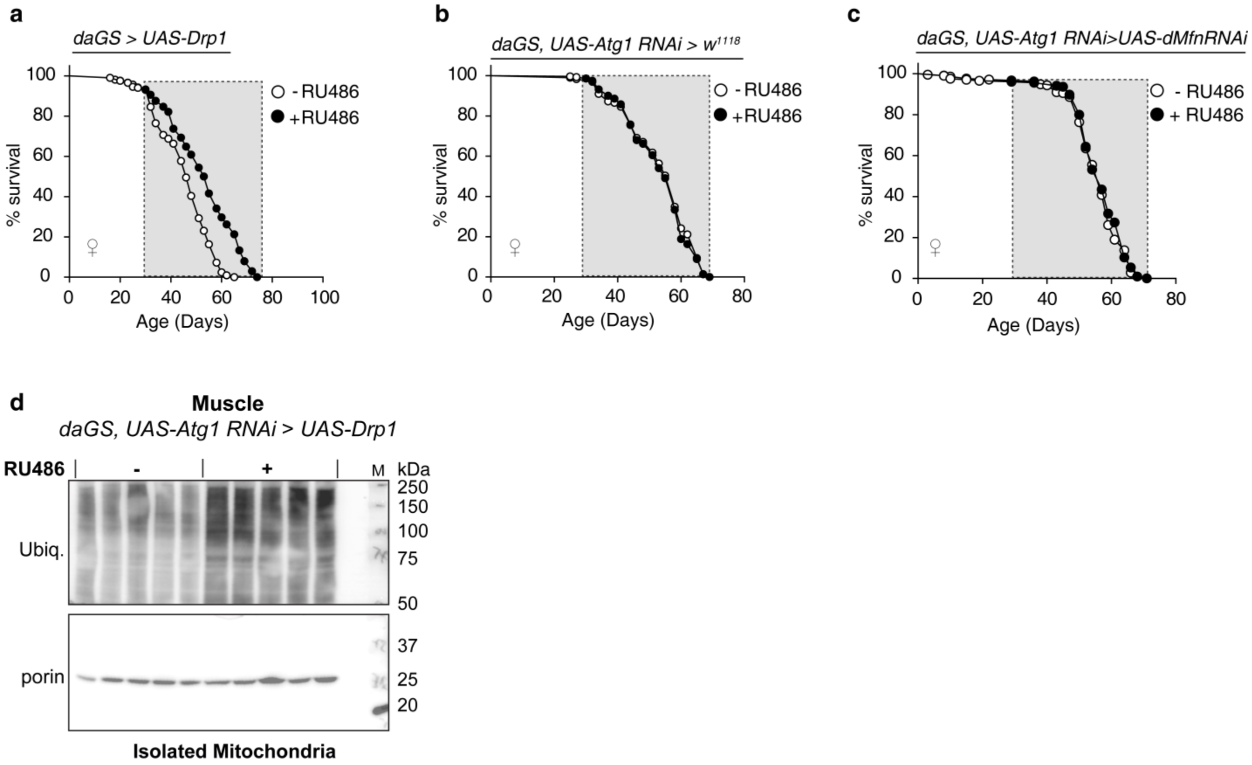

(a) Survival curves of *daGS>UAS-Drp1* females with or without RU486-mediated transgene induction from day 30 onwards. The shaded area indicates the duration of *Drp1* induction. p<0.0001, log rank test; n > 208 flies.

(b) Survival curves of *daGS, UAS-Atg1 RNAi> w^{1118}* with or without RU486-mediated transgene induction since midlife, from day 30 onwards, of *Drosophila* lifespan. The shaded area indicates the duration of *Atg1RNAi*. p = non-significant, log rank test; n > 236 flies.

(c) Survival curves of *daGS, UAS-Atg1 RNAi>UAS-dMfnRNAi* with or without RU486-mediated transgene induction since midlife, from day 30 onwards, of *Drosophila* lifespan. The

shaded area indicates the duration of *Atg1RNAi and dMfnRNAi*. p = non-significant, log rank test; n > 258 flies.

(d) Western blot detection of total ubiquitin-conjugated proteins in isolated mitochondria on day 37 in *daGS, UAS-Atg1 RNAi>UAS-Drp1* females with or without RU486-mediated transgene induction from day 30 to 37.

RU486 was provided in the media at a concentration of 25μg/ml.

Supplementary Figure 8. Validation of anti-Atg8a and anti-P62

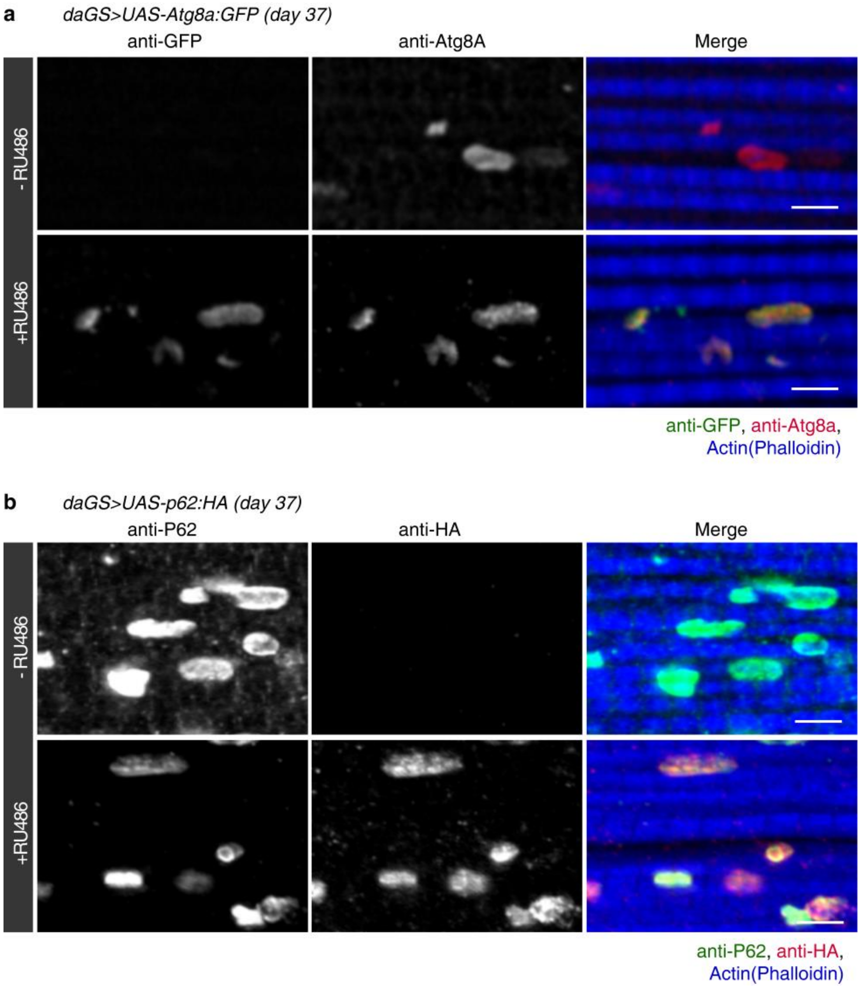

(a) Immunostaining of indirect flight muscles from day 37 daGS>*UAS-Atg8a*:GFP females with or without RU486 feeding from day 30 to day 37, showing Atg8a:GFP (green channel, anti-GFP), Atg8a (red channel, anti-Atg8a) and muscles (blue channel, stained with Rhodamine Phalloidin). Scale bar is 5µm.

(b) Immunostaining of indirect flight muscles from day 37 daGS>*UAS-p62*:HA females with or without RU486 feeding from day 30 to day 37, showing P62 staining (green channel, anti-P62), P62:HA (red channel, anti-HA) and muscles (blue channel, stained with Rhodamine Phalloidin). Scale bar is 5µm.

RU486 was provided in the media at a concentration of 50 μg/ml.

Supplementary Table 1. Lifespan information associated with Figure 1.

Genotype	Time of RU486 feeding	Lifespans	Sex	Sample size	RU486 dose in µg/ml of food	RU486 -VE	RU486 +VE	% change in median lifespan	Log Rank p-value
daGS>UAS Drp1	Midlife	1e	F	>179	25	51	60	18	<0.0001
	Midlife	S1f	F	>270	25	47	57	21	<0.0001
	Midlife	Repeat	F	>149	25	57	68	19	<0.0001
	Midlife	S1g (construct II)	F	>177	25	67	74	10	<0.0001
	Midlife	S1e	M	>146	5	55	60	9	<0.0001
	Midlife	Repeat	M	> 172	5	54	58	7	<0.0001
	Transient	S1l	F	>182	25	52	61	17	<0.0001
	Transient	1g	F	>291	25	47	57	21	<0.0001
	Adulthood	1	F	>300	25	47	50	6	0.0295
	Adulthood	Repeat	F	>167	25	45	47	4	0.8053
	Adulthood	Repeat	F	>257	25	52	52	0	0.7826
daGS>UAS $Drp1^{K38A}$	Midlife	S1h	F	>208	25	50	43	-14	<0.0001
	Midlife	Repeat	F	>188	25	50	43	-14	<0.0001
daGS>UAS dMfn	Midlife	S1i	F	>181	25	52	50	-4	<0.0001
	Midlife	Repeat	F	>199	25	54	52	-4	<0.0001
ELAVGS>UAS Drp1	Midlife	S1j	F	>152	25	39	42	8	<0.0001
	Midlife	Repeat	F	> 176	25	37	40	8	<0.0001
5966GS>UAS Drp1	Midlife	S1k	F	>225	10	65	72	11	<0.0001
	Midlife	Repeat	F	> 181	10	65	71	9	<0.0001
daGS>UAS dMfnRNAi	Midlife	1f	F	>175	50	50	57	14	<0.0001
	Midlife	Repeat	F	>208	50	52	57	10	<0.0001

Median lifespan change is shown as percentages.
